# *Argania Spinosa* Fruit Shell Extract-Induced Melanogenesis via cAMP Signaling Pathway Activation

**DOI:** 10.3390/ijms21072539

**Published:** 2020-04-06

**Authors:** Rachida Makbal, Myra O. Villareal, Chemseddoha Gadhi, Abdellatif Hafidi, Hiroko Isoda

**Affiliations:** 1 Alliance for Research on the Mediterranean and North Africa (ARENA), University of Tsukuba, Tennodai 1-1-1, Tsukuba City, Ibaraki 305-8572, Japan; makbal.rachida@gmail.com (R.M.); villareal.myra.o.gn@u.tsukuba.ac.jp (M.O.V.); 2 Faculty of Sciences Semlalia, Cadi Ayyad University, Avenue Prince Moulay Abdellah, BP 2390, Marrakesh 40000, Morocco; a.hafidi@uca.ac.ma; 3 Faculty of Life and Environmental Sciences, University of Tsukuba, Tennodai 1-1-1, Tsukuba City, Ibaraki 305-8572, Japan

**Keywords:** B16F10 cells, pigmentation, MITF, MAPKs, argan fruit shell

## Abstract

We have previously reported that argan oil and argan press-cake from the kernels of *Argania spinosa* have an anti-melanogenesis effect. Here, the effect of argan fruit shell ethanol extract (AFSEE) on melanogenesis in B16F10 cells was determined, and the mechanism underlying its effect was elucidated. The proliferation of AFSEE-treated B16F10 cells was evaluated using the 3-(4,5-dimethylthiazolyl-2)-2,5-diphenyltetrazolium bromide (MTT) assay, while the melanin content was quantified using a spectrophotometric method. The expression of melanogenesis-related proteins was determined by Western blot and real-time PCR, while global gene expression was determined using a DNA microarray. In vitro analysis results showed that the melanin content of B16F10 cells was significantly increased by AFSEE, without cytotoxicity, by increasing the melanogenic enzyme tyrosinase (TRY), tyrosinase related-protein 1 (TRP1), and dopachrome tautomerase (DCT) protein and mRNA expression, as well as upregulating microphthalmia-associated transcription factor (MITF) expression through mitogen-activated protein kinases (MAPKs) extracellular signal-regulated kinase (ERK) and p38, and the cyclic adenosine monophosphate (cAMP) signaling pathway, as indicated by the microarray analysis results. AFSEE’s melanogenesis promotion effect is primarily attributed to its polyphenolic components. In conclusion, AFSEE promotes melanogenesis in B16F10 cells by upregulating the expression of the melanogenic enzymes through the cAMP–MITF signaling pathway.AFSEE may be used as a cosmetics product component to promote melanogenesis, or as a therapeutic against hypopigmentation disorders.

## 1. Introduction

Cutaneous pigmentation is the primary defense of the human skin against harmful UV radiation, and it is imparted by the pigment melanin, a polymer resulting from melanogenesis in melanocytes [1]. Defects in melanocyte functions can lead to the loss of melanin pigment, bringing about hypopigmentation disorders, such as progressive macular hypomelanosis. At present, the treatment for hypopigmentation involves the use of topical corticosteroids, laser treatment, or skin surgery, which often have unwanted side effects. Thus, the discovery of natural compounds that can stimulate melanogenesis would be an interesting alternative to popular but carcinogenic means of promoting pigmentation.

Melanogenesis is a multi-step biosynthetic process catalyzed by tyrosinase (TYR), tyrosinase-related protein-1 (TRP1), and dopachrome tautomerase (DCT). TYR catalyzes the hydroxylation of L-tyrosine to 3,4-dihydroxyphenylalanine (L-DOPA), and the oxidation of L-DOPA to DOPA-quinone. DCT catalyzes the rearrangement of DOPA-chrome to dihydroxyindole-2-carboxylic acid (DHICA), while TRP-1 oxidizes DHICA to a carboxylatedindole-quinone that polymerizes into melanin [2]. These enzymes are under the transcriptional regulation of the microphthalmia-associated transcription factor (MITF),which in turn is regulated by a number of signaling pathways that include the cyclic adenosine monophosphate (cAMP) and Wnt signaling pathways [3]. cAMP is one of the key factors involved in the signal transduction pathways that regulate melanogenesis. The principal intracellular target of cAMP in mammalian cells is protein kinase A (PKA), which phosphorylates serine and threonine residues on target proteins, such as the cAMP responsive element binding protein (CREB) and CREB-binding protein (CBP). Phosphorylated CREB interacts with CBP to activate MITF [4].

*Argania spinosa* oil and argan press-cake have been reported to regulate melanogenesis [5,6]. However, there are no studies that have examined the potential of argan fruit shell in regulating melanogenesis. Argan fruit shell contains procyanidin, phloridzin, epicatechin, rutin, and isoquercitrin, among others [7], some of which have been reported to regulate melanogenesis [8]. Thus, in the current study, we determine the regulatory effect of argan fruit shell ethanol extract (AFSEE) on melanogenesis using B16F10 melanoma cells.

## 2. Results

### 2.1. AFSEE Has No Cytotoxic Effect on B16F10 Cells

**Figure 1:**
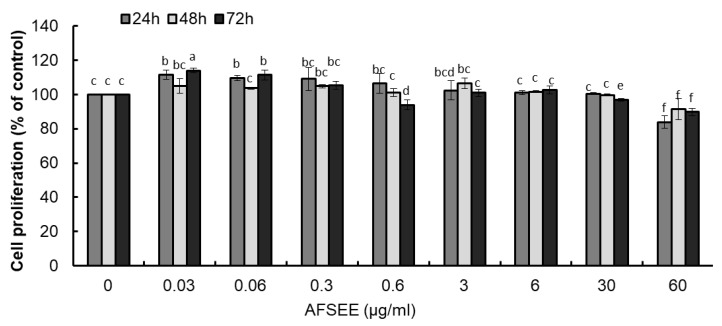
Effect of argan fruit shell ethanol extract (AFSEE) on B16F10 cell proliferation, determined using the 3-(4,5-dimethylthiazolyl-2)-2,5-diphenyltetrazolium bromide (MTT) assay. B16F10 cells were treated with AFSEE (0–60 µg/mL) for 24 h, 48 h, or 72 h. Each bar represents the percentage of viable cells relative to the control, expressed as mean ± standard deviation (SD) of four independent experiments, each one performed in triplicate. Data was subjected to ANOVA (*n* = 3). All comparisons were made between treatments. Different letters indicate treatment differences at the *p* ≤ 0.05 level.

The cytotoxicity of a drug is of chief importance when it is used, either as a medicine or as a cosmetic agent [9]. The results of the evaluation of different doses of AFSEE on cell proliferation (24 h, 48 h, and 72 h treatment) showed that the proliferation of B16F10 cells was not affected by AFSEE in a dose- and time-dependent manner (Figure 1). AFSEE at 6 and 30 μg/mL were considered to be optimum concentrations for use in the investigation of AFSEE’s effecton melanogenesis and in elucidating the molecular mechanism underlying its effect.

### 2.2. AFSEE Enhances Melanogenesis in B16F10 Cells

**Figure 2:**
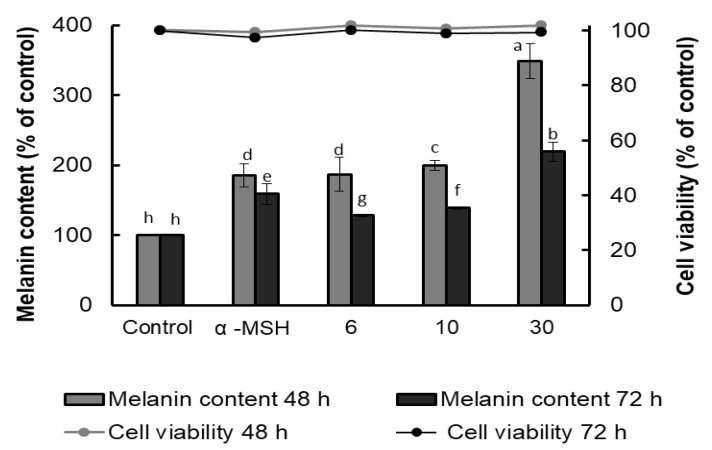
Effect of argan fruit shell ethanol extract (AFSEE) on the melanin content (bar graph) and cell viability (line graph) of B16F10 cells cultured in a 100 mm dish at a density of 5 × 10^5^ cells/dish, and treated without (control) or with α-MSH (200 mM) or AFSEE (6, 10, and 30 µg/mL) for 48 or 72 h. Each bar represents the percentage of viable cells versus control, expressed as mean ± SD of four independent experiments, each one performed in triplicate. Data were subjected to ANOVA (*n* = 3). All comparisons were made between treatments. Different letters indicate treatment differences at *p* ≤ 0.05 level.

The melanin content of B16F10 cells was evaluated using α-MSH as a positive control, and the results showed that AFSEE promoted melanogenesis in a dose-dependent manner. Compared to α-MSH, surprisingly, AFSEE-treated cells had a higher melanin content (185%) (Figure 2). Treatment with AFSEE for 48 h significantly increased the melanin content of B16F10 cells to 349%, compared to the control without cytotoxicity, while α-MSH increased the melanin content to 185%. Increasing the treatment time to 72 h also showed an increase in the melanin content (159% and 219% in α-MSH- and AFSEE-treated cells, respectively), but not after only 48 h. The highest concentration of AFSEE that promoted melanin synthesis without cytotoxicity was 30 µg/mL.

### 2.3. Melanogenic Enzymes Expression Level in B16 Melanoma Cells

**Figure 3:**
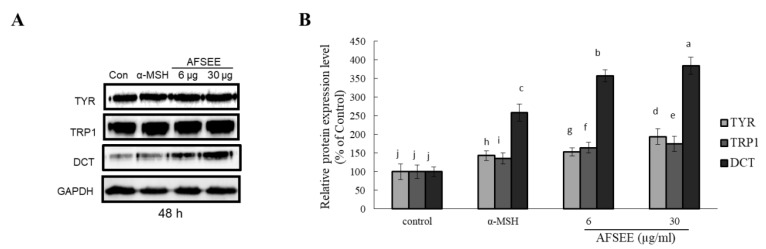
Effect of argan fruit shell ethanol extract (AFSEE) on the expression level of the melanogenic enzymes tyrosinase (TYR), tyrosinase-related protein 1 (TRP1), and dopachrome tautomerase (DCT). (**A**) The expression level of TYR, TRP1, and DCT was determined by Western blotting. B16F10 cells were cultured in a 100 mm dish at a density of 3 × 10^5^ cells/dish, and treated without (control) or with α-MSH (200 mM) or AFSEE (6 µg/mL and 30 µg/mL) for 48 h. (**B**) The protein band intensities of TYR, TRP1, and DCT were obtained using Li-COR Software. Data were subjected to ANOVA (*n* = 3). All comparisons were made between treatments. Different letters indicate treatment differences at the *p* ≤ 0.05 level.

In order to clarify the mechanism underlying the observed AFSEE-induced melanogenesis, the expression of melanogenic enzymes TYR, TRP1, DCT, and their respective intensities (Figure 3) were determined. AFSEE (30 µg/mL) significantly increased the expression level of TYR, TRP1, and DCT by 194%, 175%, and 384%, respectively, compared to the control. The significant effect on enzyme expression was observed after 48 h of treatment; α-MSH, as expected, also enhanced melanogenic enzyme expression, but not as much as AFSEE (Figure 3). A time-dependent effect (12 h, 24 h, 48 h, and 72 h) on the melanogenesis promotion of AFSEE was also observed (Supplementary Figure S1).

### 2.4. AFSEE Inhibited MITF Phosphorylation in B16F10 Cells

**Figure 4:**
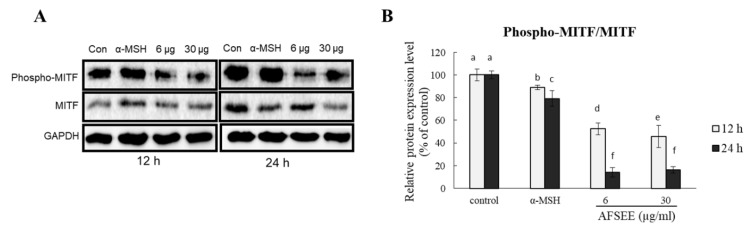
Effect of argan fruit shell ethanol extract (AFSEE) on the expression level of phosphorylated microphthalmia-associated transcription factor (pMITF) and total MITF. (**A**) The expression level of pMITF and MITF were determined by Western blotting. B16F10 cells were cultured in a 100 mm dish at a density of 3 × 10^5^ cells/dish and treated without (control) or with α-MSH (200 mM) or AFSEE (6 µg/mL and 30 µg/mL) for 12 h and 24 h. (**B**) The protein band intensities of pMITF and MITF were obtained using Li-COR Software. Data were subjected to ANOVA (*n* = 3). All comparisons were made between treatments. Different letters indicate treatment differences at the *p* ≤ 0.05 level.

To understand the mechanism underlying the effect of AFSEE on melanogenesis, we determined the effect AFSEE on MITF phosphorylation. AFSEE (30 μg/mL) significantly decreased MITF phosphorylation (Figure 4). AFSEE-decreased MITF phosphorylation was observed after 12 h and 24 h (54% and 84%, respectively (*p* ≤ 0.05)).

### 2.5. Influence of AFSEE on ERK and p38 Mitogen-Activated Protein Kinases in B16F10 Cells

**Figure 5:**
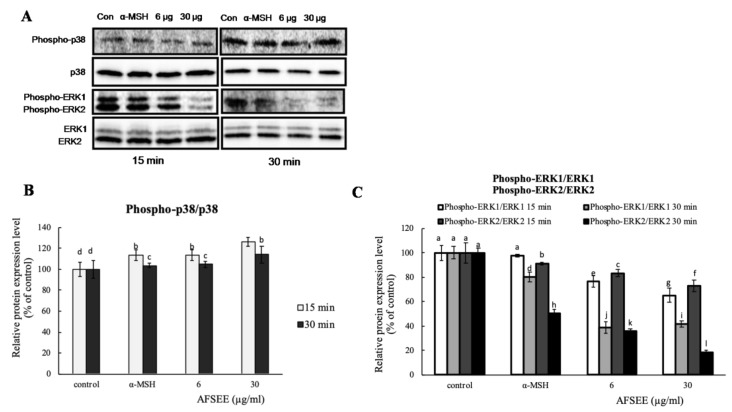
Effect of argan fruit shell ethanol extract (AFSEE) on the expression level of mitogen-activated protein kinases (MAPKs). (**A**) The expression level of phosphorylated MAPK p38 (phospho-p38), total p38 MAPK (p38), phosphorylated extracellular signal-regulated kinase 1/2 (pERK1/2), and ERK1/2 were determined by Western blotting. B16F10 cells were cultured in 100 mm dish at a density of 3 × 10^5^ cells/dish and treated without (control) or with α-MSH (200 mM) or AFSEE (6 µg/mL and 30 µg/mL) for 15 min and 30 min. (**B**, **C**) The protein band intensities of p38 and ERK1/2 were obtained using Li-COR Software. Data were subjected to ANOVA (*n* = 3). All comparisons were made between treatments. Different letters indicate treatment differences at the *p* ≤ 0.05 level.

To confirm the effect of AFSEE on mitogen-activated protein kinase (MAPK) signaling, the expression level of MAPKs p38, extracellular signal-regulated kinase 1/2 (ERK1/2) was investigated using Western blot (Figure 5). Incubation of B16F10 cells with AFSEE (6 or 30 µg/mL) for 15 min and 30 min increased the phosphorylation of p38 to 126% and 113%, respectively. A decrease in ERK1 and ERK2 phosphorylation by about 35% and 27%, respectively, was observed only at a higher AFSEE concentration (30 µg/mL), and at 15 min of AFSEE treatment. Extending the treatment time (30 min) further decreased the level of phosphorylated ERK1 (~58%) and ERK2 (~81%) (Figure 5).

### 2.6. AFSEE Upregulated Tyr, Trp1, and Dct Gene Expression

**Figure 6:**
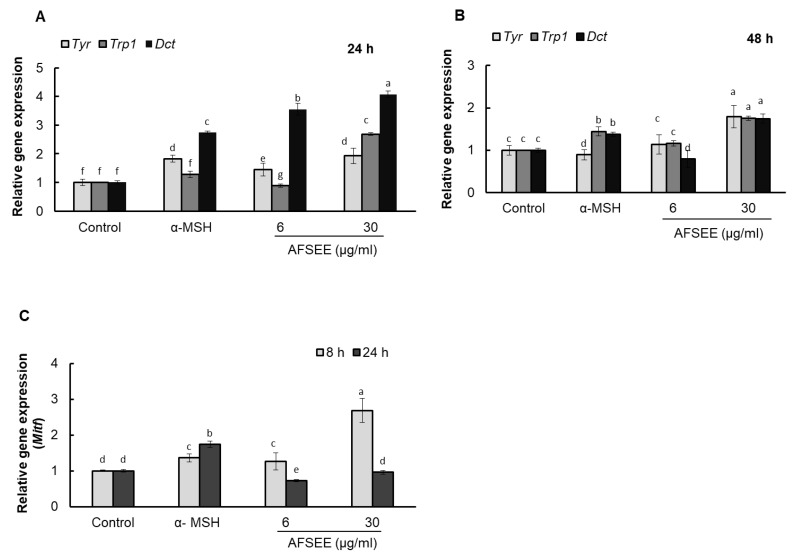
Effect of argan fruit shell ethanol extract (AFSEE) on tyrosinase (*Tyr*), tyrosinase-related protein 1 (*Trp1*), dopachrome tautomerase (*Dct*), and microphthalmia-associated transcription factor (*Mitf*) mRNA expression. B16F10 cells were cultured in a 100 mm dish at a density of 3 × 10^5^ cells/dish and treated without (control) or with α-MSH (200 mM) or AFSEE (6 µg/mL and 30 µg/mL). The expression level of *Tyr*, *Trp1,* and *Dct* mRNA expression were quantified using TaqMan real-time PCR (**A**) following treatment without (control) or with α-MSH or AFSEE for 24 h and (**B**) following treatment without or with α-MSH or AFSEE for 48 h. (**C**) The effect of AFSEE on *Mitf* expression was quantified using TaqMan real-time PCR following treatment without or with α-MSH or AFSEE for 8 h and 24 h. Data was subjected to ANOVA (*n* = 3). All comparisons were made between treatments. Different letters indicate treatment differences at the *p* ≤ 0.05 level.

Real-time PCR results showed that AFSEE increased the mRNA expression level of *Tyr*, *Trp1*, and *Dct* by 192%, 268%, and 407%, respectively, compared to the control, 24 h after treatment with 30 μg/mL AFSEE. *Tyr* expression, however, was observed to decrease when the incubation was extended by another 24 h, although the mRNA levels of AFSEE-treated cells remained higher than the control at 179%, 176%, and 174% for *Tyr*, *Trp1*, and *Dct*, respectively (Figure 6).

### 2.7. AFSEE Upregulated Mitf Expression 

AFSEE enhanced *Tyr*, *Trp1* and *Dct* gene expression, suggesting that this could be due to an upregulation of MITF. Quantification of the *Mitf* mRNA levels using real-time PCR showed a dose-dependent increase in *Mitf* levels after 8 h of AFSEE treatment, compared to the control, but was observed to decrease after 24 h of treatment (Figure 6).

### 2.8. Transcriptome Changes Induced by AFSEE in B16F10 Cells 

**Figure 7:**
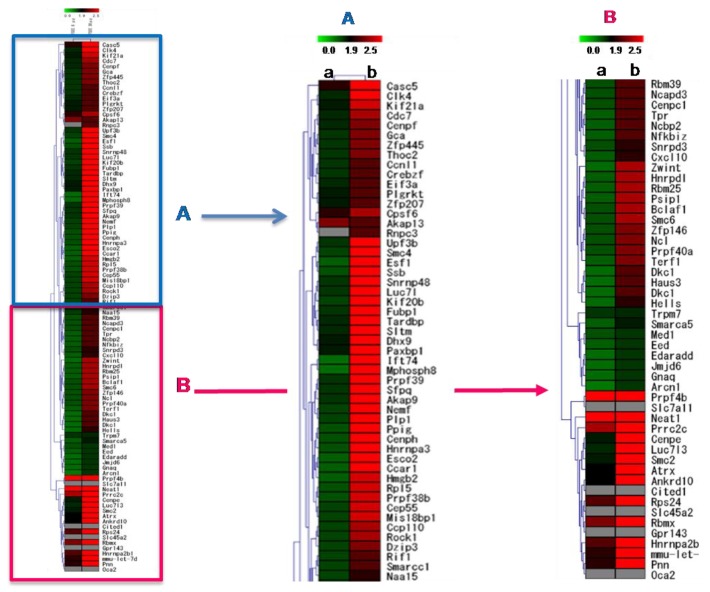
Heat map representing the effect of argan fruit shell ethanol extract (AFSEE) on global gene expression in B16F10 cells and the hierarchical clustering of genes that were differentially expressed in B16F10 cells treated without (control) or with (a) AFSEE (6 µg/mL) or (b) AFSEE (30 µg/mL) for 4 h. The 25 downregulated genes subjected to hierarchical clustering had a fold-change value of ≤ –1.5 (vs.control), while for the upregulated genes, genes with ≥ 1.5-fold change values were chosen. The map is split into two (**A**,**B**) for clarity. The Euclidian distance method was used for the comparison, and the resulting red and green colors represent gene up- and down-regulation, respectively.

Analysis of the global gene expression in B16F10 cells showed that AFSEE caused significant changes in the B16F10 gene expression profile (Figure 7). Treatment with 30 µg/mL AFSEE elicited higher gene expression fold change compared to 6 µg/mL (Table 1). Out of a total of 1700 genes that were significantly altered, 1200 genes were up-regulated (≥ 1.5-fold change) while 500 genes were down-regulated(≤ −1.5-fold change). Hierarchical clustering analysis of the top 100 genes revealed the concentration-dependent effect of AFSEE (Figure 7).

Gene ontology (GO) annotations revealed that the upregulated genes belong to multiple functional biological pathways that are significant for the positive regulation of transcription, the regulation of protein kinase activity signal transduction, and cell adhesion, while the downregulated genes were involved in pathways regulating the protein kinase cascade and cellular differentiation (Table 1).

Genes associated with pigmentation were upregulated by AFSEE. The cAMP signaling pathway-associated genes Akap13 (A-kinase anchor protein 13) and Cxcl10 (C–X–C motif chemokine ligand 10) expression were increased 2.1- and 2-fold, respectively, following treatment with 30 μg/mL of AFSEE (Table 1).

### 2.9. Validation of DNA Microarray Results by Real-Time PCR

**Figure 8:**
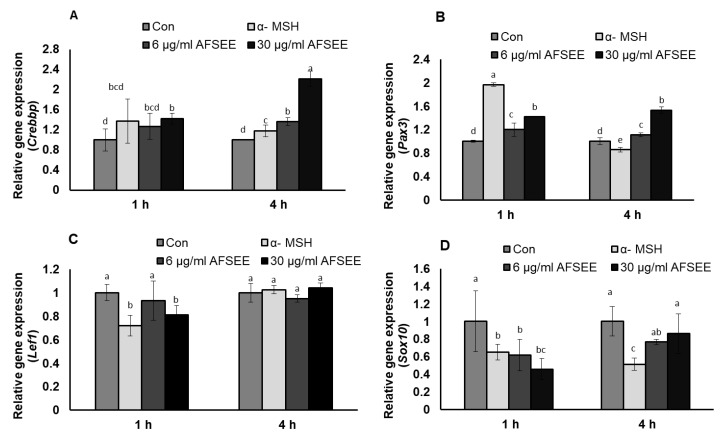
Effect of argan fruit shell ethanol extract (AFSEE) on mRNA expression of CREB binding protein (*Crebbp*), paired box gene 3 (*Pax3*), lymphoid enhancer binding factor 1 (*Lef1*), and SRY box-containing gene 10 (*Sox 10*). B16F10 cells were cultured in a 100 mm dish at a density of 3 × 10^5^ cells/dish and treated without (control) or with α-MSH (200 mM) or AFSEE (6 µg/mL and 30 µg/mL) for 1 h and 4 h (**A**) The effect of AFSEE on *Crebbp*, (**B**) *Pax3*, (**C**) *Lef1*, and (**D**) *Sox10* expression was quantified using TaqMan real-time PCR. Data were subjected to ANOVA (*n* = 4). All comparisons were made between treatments. Different letters indicate treatment differences at the *p* ≤ 0.05 level.

DNA microarray analysis showed that AFSEE modulated the expression of the genes associated with the transcription and signaling regulation of *Mitf* and *Mitf*-related signaling. Results of the validation of the DNA microarray using RT-PCR made it clear that the expression of *Mitf* transcription regulators *Crebbp, Pax3, Lef1*, and *Sox10* was indeed changed by AFSEE in a time-dependent manner (Figure 8). *Crebbp* was increased 2.2-fold, while *Pax3* expression was increased 1.5-fold. Treatment with AFSEE for 4 h did not affect the expression of *Lef1* (1-fold), but it downregulated *Sox10* expression by 0.8-fold.

### 2.10. Phytochemical Characterization of AFSEE

The qualitative phytochemical screening of AFSEE revealed the presence of polyphenols (flavonoids, tannins and coumarins), the presence of saponins, and the absence of alkaloids (Table 2). The spectrophotometric quantification of the two main families of secondary metabolites detected in AFSEE showed that the AFSEE polyphenol and saponin content was 22.1 ± 0.87 mg gallic acid equivalent/g dry weight and 16.23 ± 0.13 mg oleanolic acid equivalent/g dry weight, respectively.

**Figure 9:**
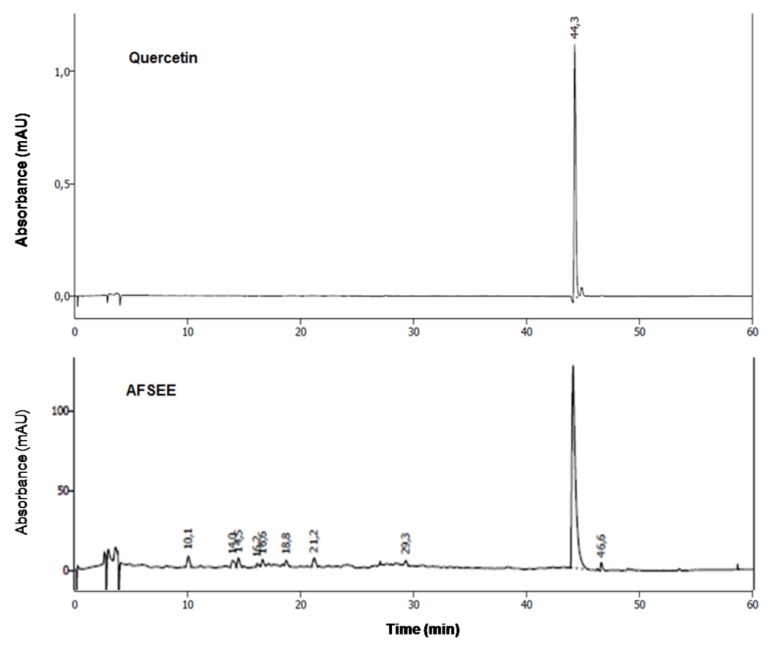
High-performance liquid chromatography (HPLC) fingerprinting of AFSEE: chromatograms of AFSEE and quercetin, AFSEE’s major polyphenol compound, were acquired at 280 nm. HPLC chromatograms of standards used in the analysis are included as supplementary figures.

The chemical characterization of AFSEE polyphenols was performed by HPLC, by comparison of the retention times and the UV spectrum of AFSEE peaks with those of reference standards. The results demonstrate that quercetin is the major phenolic compound in AFSEE (Figure 9, Supplementary Figure S2).

## 3. Discussion

Melanin is a photoprotective skin pigment that plays a critical role in protecting human skin from the harmful effects of UV radiation, and pathologies characterized by hypo- or hyperpigmentation are common. Melanin biosynthesis in the skin is initiated upon exposure to UV radiation, promoting the expression of the proopiomelanocortin (POMC) protein in the keratinocytes [10], which causes α-MSH to cleave from its precursor POMC protein [11] and bind to the receptor MC1R, activating adenylyl cyclase that will generate cAMP [12]. The accumulation of cAMP activates PKA, causing phosphorylation of the cAMP responsive binding element (CREB), which in turn promotes the transcription of the microphthalmia transcription factor (MITF). MITF acts as the master regulator of the expression of multiple enzymes that catalyze melanin biosynthesis, including tyrosinase (TYR), tyrosinase-related protein 1 (TRP1), and dopachrome tautomerase (DCT) [13]. Previous studies have indicated that the activation of MAP kinase family members ERK, c-Jun N-terminal kinase (JNK), and p38 plays an important role in MITF regulation [14,15,16]. MITF contains a basic helix–loop–helix–leucine zipper domain in its structure [13], and specifically binds to the M-box and E-box motifs in the promoter regions of *Tyr*, *Trp1*, and *Dct* to regulate their expression [17]. Within this context, natural compounds that affect the expression or activity of these enzymes or MITF may be considered as potential therapeutics for the regulation of pigmentation or treatment of pigmentary disorders [18]. In this study, we evaluated the effect of argan fruit shell ethanol extract (AFSEE) on melanogenesis in B16F10 cells. In order to avoid interference in melanogenesis caused by the reduction of cell numbers, due to the testing extract, only non-toxic concentrations of AFSEE (6 µg/mL to 30 µg/mL) were used in the experiments. AFSEE significantly increased the melanin content in B16F10 cells in a dose- and time-dependent manner (Figure 2), and this effect may be attributed to the flavonoid content of argan fruit shells; since quercetin which was identified as the major polyphenol compound in AFSEE (Figure 9), it has been reported to be an enhancer of melanin in human melanoma cells [19]. Argan fruit shell also contains bidesmosidic saponins with oleanane-type aglycone, namely misaponin A, arganin M, and arganin N [20,21]. Oleanane-type saponins have been reported as inhibitors of melanogenesis [21]. It could therefore be assumed that the melanogenesis promotion effect of AFSEE is a result of the synergistic effects of these compounds.

Treatment with AFSEE for 48 h increased the expression level of TYR, TRP1, and DCT in a dose-dependent manner, demonstrating that the AFSEE-induced melanogenesis was primarily due to the elevated TYR, TRP-1, and DCT expression (Figure 3). It appears that 48 h of treatment is the most effective treatment time, since incubation with AFSEE at 12 h, 24 h, and 72 h was not as effective as at 48 h (Supplementary Figure S1). This is what was observed as the effect of AFSEE on the melanogenic enzymes, with regards to treatment time. The increased melanogenic enzyme expression was caused by the inhibition of MITF phosphorylation (Figure 4). MITF phosphorylation leads to ubiquitination and proteasome-mediated degradation of MITF [22], suggesting that the inhibition of MITF phosphorylation plays an important role in AFSEE-induced melanogenesis promotion, and the time when the melanogenic enzymes expression is affected will depend on when the MITF is activated. Other natural products or plant extracts were observed to have different effects on MITF, depending on treatment time. In one of our previous reports, transcription factor *Mitf* RNA expression may be observed after 4 h [23]. In another, the significant effect of hirsein A from *T. hirsuta* on MITF (protein expression) was observed after 24 h and 48 h [5].

The MAPK family includes extracellular responsive kinase (ERK), c-Jun N-terminal kinase (JNK), and p38 MAPK [24]. p38 MAPK is a major intracellular signaling molecule critical to pigmentation, as its activation is associated with increased melanin synthesis, while activation of ERK 1/2 and JNK is associated with decreased melanogenesis [14]; p38 MAPK signaling has been reported to activate MITF [25], while ERK activation leads to MITF degradation [26]. Argan oil has been demonstrated to promote MITF degradation, as we have previously reported [5], and which the work of Caporarello et al. [27] on uveal melanocytes has corroborated. The MITF phosphorylation was inhibited by AFSEE (Figure 4). Moreover, AFSEE significantly decreased ERK1 and ERK2 phosphorylation (Figure 5). MITF appears to be regulated through the MAPK pathway, but also by the cAMP pathway [3]. The cAMP pathway plays a key role in the regulation of melanogenesis at the transcription and translation levels [28]. The cAMP pathway activates CREB, which binds to the MITF promoter; at the same time, cAMP activates MAPK, which will also activate MITF. At the transcriptional level, AFSEE enhanced the expression of *Mitf*, and as a result, the melanogenic enzymes (Figure 6). DNA microarray analysis of AFSEE-treated B16F10 cells showed significant changes in the expression of genes associated with the cAMP signaling pathway and in the modulation of MITF transcriptional regulators *Pax3* and *Crebbp* (Figure 8). *Crebbp* encodes for the protein CREBBP and binds to phosphorylated CREB to enhance its activity [4]. Activation of p38 has been shown to contribute positively to melanogenesis by activating CREB. In addition, the activation of CREB-binding protein is required for cAMP responsiveness of the MITF promoter [29]. Interestingly, based on the DNA microarray results, among all the genes modulated by AFSEE (Table 1), cAMP signaling pathway genes *Akap13* and *Cxcl10* were upregulated, and could have contributed to the overall effect of AFSEE. *Akap13* plays a critical role in coordinating cAMP signaling in adrenocortical cells [30], while *Cxcl10* increases the level of activated CREB [31]. Therefore, the AFSEE-enhanced *Crebbp* expression activated MITF and led to the promotion of melanin synthesis in B16F10 cells.

## 4. Materials and Methods

### 4.1. Reagents

For cell culture, RPMI 1640 medium (Gibco, Thermo Fisher Scientific, Waltham, MA, USA), fetal bovine serum (FBS;Bio West, Miami, FL, USA), alpha-melanocyte-stimulating hormone (α-MSH; Sigma Aldrich, St. Louis, MO, USA), phosphate-buffered saline (PBS; SigmaChemical Co., Deisenhofen, Germany), and trypsin/EDTA (0.25% trypsin/0.02% EDTA in PBS; Sigma Chemical Co., Deisenhofen, Germany) were used.

The MTT assay used 3-(4,5-dimethylthiazolyl-2)-2,5-diphenyltetrazolium bromide (MTT; Dojindo, Japan), sodium diodecyl sulfate (SDS; WAKO, Osaka, Japan), triton-x 100 (Sigma Aldrich, St. Louis, MO, USA), trichloroacetic acid (TCA; Wako, Japan), and 8N sodium hydroxide solution (NaOH; Wako, Japan).

The Western blot analysis used resolving gel buffer 0.5 and 1.5M Tris-HCL (pH=8.8, BIO-RAD, Hercules, CA, USA), acrylamide/Bis mixed solution (BIO-RAD, Hercules, CA, USA), TEMED (GE Healthcare, Buckinghamshire, UK), mercapto-ethanol (Sigma Aldrich, St. Louis, MO, USA), ammonium persulfate (GE Healthcare, Uppsala, Sweden), tween 20 (Sigma, Germany), and Odyssey Blocking buffer (Li-Cor Biosciences, Lincoln, Nebraska, USA).

The real-time PCR included an ISOGEN kit (Nippon Gene, Tokyo, Japan), chloroform (Wako, Japan), 2-propanol (Molecular Biology, Wako, Japan), ethanol 99.5% (Wako, Japan), tris-EDTA buffer solution (Fluka, Japan), and TaqMan Gene Expression Master Mix (Applied Biosystems, United States).

Lastly, for HPLC, acetonitrile, quercetin, rutin, catechin, epicatechin, caffeic acid, 3–4 dihydroxybenzoïc acid, chlorogenic acid, p-coumaric acid, gallic acid, and rosmarinic acid standards, were all from Sigma (Germany).

### 4.2. Preparation of Argan Fruit Shell Extract

Powdered argan fruit shells (10 g) were extracted using 100 mL ethanol (70%) in the dark at room temperature for two weeks. The extraction yield was 3.5%. Argan fruit shell ethanol extract (AFSEE) was filter sterilized using 0.22 µm filter (Millex GV, Millipore, Bedford, USA) and stored at −80 °C until use.

Argan (*Argania spinosa*) drupe-like fruits, containing the shell, were collected in June 2016 from Mirght (Sidi Ifni region), in the southwestern part of Morocco, and were authenticated by Prof. Ahmed Ouhammou from Cadi Ayyad University, Faculty of Sciences Semlalia, Department of Biology, Marrakech, Morocco. A voucher specimen of plant material (MARK 10888) was deposited in the Herbarium of the same institution

### 4.3. AFSEE Chemical Characterization

AFSEE was screened for the presence of alkaloids, flavonoids, coumarins, tannins, and saponins. The qualitative determination of these phytochemicals was conducted using previously reported methods [32,33].

The AFSEE total phenol content, flavonoid content, condensed tannins, and saponin content were determined according to methods previously reported [34,35,36,37]. They were expressed as mg of gallic acid equivalents per g of dry weight, mg of catechin equivalent per g of dry weight, mg of catechin equivalent per g of dry weight, and mg of oleanolic acid equivalent per g of dry weight, respectively.

The chemical characterization of AFSEE was performed using high-performance liquid chromatography (HPLC; Knauer; Germany), equipped with a smart line pump (K-1001) and a photometric diode array (PDA) detector 28000 (200-700 UV-Vis) operating at 280 nm. The device was equipped with a Eurospher II 100-5 C 18 (250 × 4.6 mm) column, and the temperature was maintained at 25 °C. A mixture of (A) acidified water (pH 2.6) (A) and acetonitrile (B) was used as a mobile phase, for a total running time of 60 min.The flow rate was 1 mL/min with gradient program (0–54 min, 5–100% B). The sample volume injected was 10 μL. The tentative identification of phenolic compounds in AFSEE was performed by a comparison of retention times and UV-visible spectra of unknown peaks with the standards (quercetin, rutin, catechin, epicatechin, caffeic acid, 3-4 dihydroxibenzoïc acid, chlorogenic acid, p-coumaric acid, gallic acid, and rosmarinic acid).

### 4.4. Cell Culture 

Murine melanoma cell line B16F10 (RCB2630) was purchased from Riken Cell Bank in Tsukuba, Japan. Melanoma cells were maintained as a monolayer culture in RPMI 1640 medium, supplemented with 10% FBS and incubated at 37 °C in a humidified atmosphere of 5% CO_2_. 

### 4.5. Cell Proliferation

The effect on B16F10 cells proliferation was evaluated using the MTT assay, as we have previously reported [38]. The B16F10 cells were treated with various concentrations of AFSEE (0, 0.03, 0.06, 0.3, 0.6, 3, 6, 30, 60 μg/mL) for 24 h, 48 h, or 72 h.

### 4.6. Melanin Quantification

The melanin content of the B16F10 cells treated with or without AFSEE was quantified spectrophotometrically, as previously reported [6]. B16F10 cells (5 × 10^4^ cells/mL) were treated without (control), or with α-MSH (200 mM) (positive control) or AFSEE (6, 10 and 30 μg/mL) for 48 h and 72 h. Cells lysates were centrifuged at 3000 rpm for 5 min after 2 h (room temperature) heating in NaOH. All samples were assayed in triplicates. 

### 4.7. Western Blot

The total protein (10 μg) extracted from the B16F10 cells treated without (control) or with α- MSH (200 mM) or AFSEE (6 or 30 μg/mL) at different time points was separated, following the manufacturer’s instructions for BIO RAD Mini-Protean II for 10% gel preparation using 30% acrylamide gel and blotted onto PVDF membrane (Millipore, Germany). The blots were probed with primary antibodies for MITF, phosphorylated MITF, TYR, TRP1, DCT, MAPKs (phosphorylated-p38, p38, phosphorylated ERK1/2, ERK1/2), and GAPDH. The proteins were visualized using a LiCor Odyssey Infrared Imaging System (LI-COR Biosciences, United States) after reaction with goat anti-mouse IRDye 680LT or goat anti-rabbit IRDye 800CW (LI-COR). Antibodies were diluted as recommended by the manufacturer, and in case there were none, the antibodies were diluted 1/500 *v*/*v* for primary antibodies and 1/1000 *v*/*v* for the secondary antibodies.

### 4.8. Real-Time PCR

RNA from samples was extracted using ISOGEN (Wako, Japan) and used as templates for the reverse transcription polymerase chain reaction (RT-PCR), using SuperScript III reverse transcriptase kit (Invitrogen, Carlsbad, CA, United States), following the manufacturer’s instructions. Real–time PCR analysis (RT-PCR) was performed in 7500 Fast Real-time PCR (Applied Biosystems, United States). Real-time Primers for *Mitf* (Mm0043495-m1), *Tyr* (Mm00495817-m1), *Trp1* (Mm00453201-m1), *Dct* (Mm01225584-m1), *Crebbp* (Mm01342452-m1), *Pax3* (Mm00435491-m1), *Lef1* (Mm00550265-m1), and *Sox10* (Mm00569909-m1) were used (Applied Biosystems, Foster City, CA, United States). *Gapdh* (Mm99999915-g1) was used as an endogenous control. The thermal cycling protocol was as follows: 95 °C for 10 min, followed by 40 cycles of 95 °C for 15 s and 60 °C for 1 min.

### 4.9. DNA Microarray Analysis

The global gene expression changes in B16F10 cells were analyzed following the protocol for the Affymetrix MG-430 PM Array Strip (Affymetrix, Santa Clara, CA, USA). Partek Express Software (Affymetrix) was used to analyze the data, by running comparisons of gene expression in treated and control cells based on mathematical algorithms. The generated data (significant fold change in gene expression) was then analyzed using the Pathway Studio Explore 1.1 software (Affymetrix). The DNA microarray data comply with MIAME guidelines, and have been deposited in the ArrayExpress database at EMBI-EBI (www.ebi.ac.uk/arrayexpress), under the reference number E-MTAB-8836.

### 4.10. Statistical Analysis

The results are expressed as mean ± standard deviation (SD) of at least three independent experiments. The data were statistically analyzed using one-way analysis of variance, with a Fisher’s least significant difference (LSD) post-hoc test, using CoStat 6.451 software, and two-way ANOVA, followed by a Tukey post-hoc test, using IBM SPSS statistics software. From these, *p*-values less than 0.05 were considered to be statistically significant.

## 5. Conclusions

The differentiation (or melanin biosynthesis) and associated signaling mechanism of melanoma cells (i.e., B16 cell line) and melanocytes (pigment cells) have been discussed by Bennett [39]. Both melanoma cells and melanocytes require the activation of tyrosinase (TYR) to produce melanin, and α-MSH and other inducers may be used to activate TYR. In this study, AFSEE has been demonstrated to promote melanogenesis in B16F10 cells by its effect on the melanogenic enzymes, through the cAMP–MITF signaling pathway. A follow-up study using human epidermal melanocytes and animal models may be necessary to demonstrate its effect on human skin cells, and as a preliminary step before evaluation as an active component of a cosmetic or therapeutics designed to promote melanogenesis.

## Figures and Tables

**Table 1:** List of up- and down-regulated genes in B16F10 melanoma cells treated with AFSEE (6 µg/mL and 30 µg/mL), as determined by DNA microarray.

Gene Symbol	Gene Title	Biological Process	AFSEE 6 µg/mL	AFSEE 30 µg/mL
*Prpf4b*	PRP4 pre-mRNA processing factor 4 homolog B (yeast)	mRNA processing, protein phosphorylation, RNA splicing.	2.5	5.8
*Ankrd10*	Ankyrin repeat domain 10	Regulation of canonical Wnt signaling pathway.	1.9	4.0
*Cenpe*	Centromere protein E	Cell cycle, cell division, chromosome segregation, establishment of protein localization, positive regulation of protein kinase activity.	1.7	3.6
*Atrx*	Alpha thalassemia/mental retardation syndrome X-linked homolog (human)	Cellular response to DNA damage stimulus, signal transduction by p53 DNA repair, DNA replication-independent nucleosome assembly.	1.9	3.4
*Akap13*	A kinase (PRKA) anchorprotein 13	Nuclear export, regulation of cardiac muscle hypertrophy, regulation of glucocorticoid mediated signaling pathway, regulation of protein kinase activity.	2.1	2.1
*Tpr*	Translocated promoter region	Cellular response to interferon-alpha, MAPK import into nucleus, response to epidermal growth factor.	1.2	2.1
*Cxcl10*	Chemokine (C–X–C motif) ligand 10	Positive regulation of cAMP metabolic process, positive regulation of cAMP-mediated signaling, positive regulation of cell proliferation.	1.2	2.0
*Hells*	Helicase, lymphoid specific	Chromatin silencing, DNA methylation, mitotic nuclear division, multicellular organismal development.	1.1	2.0
*Med1*	Mediator complex subunit 1	Angiogenesis, keratinocyte differentiation, lactation-positive regulation of keratinocyte differentiation, ERK1 and ERK2 cascade, cellular response to epidermal growth factor stimulus, positive regulation of receptor activity, protein ubiquitination, positive regulation of protein import into nucleus, translocation.	1.3	1.5
*Edaradd*	EDAR (ectodysplasin-A receptor)-associated death domain	Cell differentiation	1.1	1.5
*Jmjd6*	Jumonji domain containing 6	Regulation of transcription, DNA-template, cell surface receptor signaling pathway.	1.1	1.5
*Eed*	Embryonic ectoderm development	Positive regulation of histone H3-K27 methylation, covalent chromatin modification.	1.2	1.5
*Gnaq*	Guanine nucleotide binding protein, alpha q polypeptide	Regulation of melanocyte differentiation, regulation of protein kinase activity, signal transduction.	1.1	1.5
*Arcn1*	Archain 1	Golgi vesicle transport, protein transport, vesicle-mediated transport.	1.0	1.5
*Slc7a11*	Solute carrier family 7 member 11	Amino acid transmembrane transport, response to toxic substance.	−1.3	−1.8
*Gpr143*	G protein-coupled receptor 143	Regulation of melanosome transport, regulation of melanosome organization.	−1.1	−2.1
*Oca2*	Oculo-cutaneous albinism II	Melanocyte differentiation, cell proliferation, transmembrane transport.	−1.3	−2.6

**Table 2:** Qualitative and quantitative phytochemical characterization of AFSEE, determined using colorimetric and spectrophotometric methods.

**Qualitative characterization**	**Parameters**	**Visual Detection**
	Alkaloids	-
	Flavonoids	+++
	Saponins	++
	Coumarins	+
**Quantitative characterization**	**Parameters**	**Content in mg/g DW**
	Total polyphenols (mg gallic acid eq/g DW)	22.11 ± 0.87
	Total flavonoids (mg catechin eq/g DW)	9.9 ± 0.2
	Condensed tannins (mg catechin eq/g DW)	1.60 ± 0.08
	Saponins (mg oleanolic acid eq/g DW)	16.23 ± 0.13

(+++) strong presence; (++) moderate presence; (+): weak presence; (-): absence; (eq): equivalent; DW: dry weight

## Supplementary Materials

Supplementary materials can be found at https://www.mdpi.com/1422-0067/21/7/2539/s1.

## Abbreviations

AFSEE	Argan fruit shell ethanol extract
Akap13	A-kinase anchor protein 13
cAMP	Cyclic adenosine monophosphate
CREB	cAMP-responsive element binding protein
Crebbp	CREB binding protein
Cxcl10	C–X–C motif chemokine ligand 10
DCT	Dopachrome tautomerase
ERK	Extracellular signal-regulated kinase
GAPDH	Glyceraldehyde 3-phosphate dehydrogenase
HPLC	High-performance liquid chromatography
Lef1	Lymphoid enhancer binding factor 1
MAPK	Mitogen-activated protein kinase
MITF	Microphthalmia-associated transcription factor
MTT	3-(4,5-dimethylthiazolyl-2)-2,5-diphenyltetrazolium bromide
Pax3	Paired box gene 3
PBS	Phosphate-buffered saline
PKA	Protein kinase A
RT-PCR	Real-time polymerase chain reaction
Sox 10	SRY-box containing gene 10
TRP1	Tyrosinase-related protein 1
TYR	Tyrosinase
α-MSH	alpha-melanocyte-stimulating hormone
α-MSH	alpha-melanocyte-stimulating hormone

## References

[1] Campos P.M., Horinouchi C.D.D.S., Prudente A.D.S., Cechinel-Filho V., Cabrini D.D.A., Otuki M.F. (2013). Effect of a *Garcinia gardneriana* (Planchon and Triana) Zappi hydroalcoholic extract on melanogenesis in B16F10 melanoma cells. J. Ethnopharmacol..

[2] Ito S. (2003). IFPCS presidential lecture: A chemist’s view of melanogenesis. Pigment Cell Res..

[3] D’Mello S.A.N., Finlay G.J., Baguley B.C., Askarian-Amiri M.E. (2016). Signaling pathways in melanogenesis. Int. J. Mol. Sci..

[4] Cardinaux J.R., Notis J.C., Zhang Q., Vo N., Craig J.C., Fass D.M., Brennan R.G., Goodman R.H. (2000). Recruitment of CREB binding protein is sufficient for CREB-mediated gene activation. Mol. Cell Biol..

[5] Villareal M.O., Kume S., Bourhim T., Bakhtaoui F.Z., Kashiwagi K., Han J., Gadhi C., Isoda H. (2013). Activation of MITF by argan oil leads to the inhibition of the tyrosinase and dopachrome tautomerase expressions in B16 murine melanoma cells. Evid. Based Complement. Alternat. Med..

[6] Bourhim T., Villareal M.O., Gadhi C., Hafidi A., Isoda H. (2018). Depigmenting effect of argan press-cake extract through the down-regulation of Mitf and melanogenic enzymes expression in B16 murine melanoma cells. Cytotechnology.

[7] El Monfalouti H., Charrouf Z., Belviso S., Ghirardello D., Scursatone B., Guillaume D., Denhez C., Zeppa G. (2012). Analysis and antioxidant capacity of the phenolic compounds from argan fruit (*Argania spinosa* (L.) Skeels). Eur. J. LipidSci. Tech..

[8] Jung E., Lee J., Huh S., Lee J., Kim Y.S., Kim G., Park D. (2009). Phloridzin-induced melanogenesis is mediated by the cAMP signaling pathway. Food Chem. Toxicol..

[9] Ko H.H., Chiang Y.C., Tsai M.H., Liang C.J., Hsu L.F., Li S.Y., Wang M.C., Yen F.L., Lee C.W. (2014). Eupafolin, a skin whitening flavonoid isolated from *Phyla nodiflora*, downregulated melanogenesis: Role of MAPK and Akt pathways. J. Ethnopharmacol..

[10] Bu J., Ma P.C., Chen Z.Q., Zhou W.Q., Fu Y.J., Li L.J., Li C.R. (2008). Inhibition of MITF and tyrosinase by paeonol-stimulated JNK/SAPK to reduction of phosphorylated CREB. Am. J. Chin. Med..

[11] Kadekaro A.L., Kanto H., Kavanagh R., Abdel-Malek Z.A. (2003). Significance of the melanocortin 1 receptor in regulating human melanocyte pigmentation, proliferation, and survival. Ann. N. Y. Acad. Sci..

[12] Solano F., Briganti S., Picardo M., Ghanem G. (2006). Hypopigmenting agents: An updated review on biological, chemical and clinical aspects. Pigment Cell Res..

[13] Levy C., Khaled M., Fisher D.E. (2006). MITF: Master regulator of melanocyte development and melanoma oncogene. Trends Mol. Med..

[14] Hirata N., Naruto S., Ohguchi K., Akao Y., Nozawa Y., Iinuma M., Matsuda H. (2007). Mechanism of the melanogenesis stimulation activity of (-)-cubebin in murine B16 melanoma cells. Bioorg. Med. Chem..

[15] Kim E.S., Park S.J., Goh M.J., Na Y.J., Jo D.S., Jo Y.K., Shin J.H., Choi E.S., Lee H.K., Kim J.Y. (2014). Mitochondrial dynamics regulate melanogenesis through proteasomal degradation of MITF via ROS-ERK activation. Pigment Cell Melanoma Res..

[16] Smalley K., Eisen T. (2000). The involvement of p38 mitogen-activated protein kinase in the alpha-melanocyte stimulating hormone (alpha-MSH)-induced melanogenic and anti-proliferative effects in B16 murine melanoma cells. FEBS Lett..

[17] Pogenberg V., Ögmundsdóttir M.H., Bergsteinsdóttir K., Schepsky A., Phung B., Deineko V., Milewski M., Steingrímsson E., Wilmanns M. (2012). Restricted leucine zipper dimerization and specificity of DNA recognition of the melanocyte master regulator MITF. Genes Dev..

[18] Abdel-Malek Z.A., Knittel J., Kadekaro A.L., Swope V.B., Starner R. (2008). The melanocortin 1 receptor and the UV response of human melanocytes—A shift in paradigm. Photochem. Photobiol..

[19] Nagata H., Takekoshi S., Takeyama R., Homma T., Osamura R.Y. (2004). Quercetin enhances melanogenesis by increasing the activity and synthesis of tyrosinase in human melanoma Cells and in normal human melanocytes. Pigment Cell Res..

[20] Alaoui A., Charrouf Z., Soufiaoui M., Carbone V., Malorni A., Pizza C., Piacente S. (2002). Triterpenoid saponins from the shells of *Argania spinosa* seeds. J. Agri. Food Chem..

[21] Akihisa T., Abe M., Manosroi J., Manosroi A. (2018). Triterpenoid saponins of Sapotaceae plants and their bioactivities. Chiang Mai J. Sci..

[22] Wu M., Hemesath T.J., Takemoto C.M., Horstmann M.A., Wells A.G., Price E.R., Fisher D.Z., Fisher D.E. (2000). c-Kit triggers dual phosphorylations, which couple activation and degradation of the essential melanocyte factor Mi. Genes Dev..

[23] Villareal M., Han J., Yamada P., Shigemori H., Isoda H. (2010). Hirseins inhibit melanogenesis by regulating the gene expressions of Mitf and melanogenesis enzymes. Exp.Dermatol..

[24] Singh S.K., Sarkar C., Mallick S., Saha B., Ber R., Bhadra R. (2005). Human placental lipid induces melanogenesis through p38 MAPK in B16F10 mouse melanoma. Pigment Cell Res..

[25] Ahn J.H., Jin S.H., Kang H.Y. (2006). LPS induces melanogenesis through p38 MAPK activation in human melanocytes. Arch. Dermatol. Res..

[26] Kim D.S., Hwang E.S., Lee J.E., Kim S.Y., Kwon S.B., Park K.C. (2003). Sphingosine-1-phosphate decreases melanin synthesis via sustained ERK activation and subsequent MITF degradation. J. Cell Sci..

[27] Caporarello N., Olivieri M., Cristaldi M., Rusciano D., Lupo G., Anfuso C.D. (2017). Melanogenesis in uveal melanoma cells: Effect of argan oil. Int. J. Mol. Med..

[28] Bertolotto C., Abbe P., Hemesath T.J., Bille K., Fisher D.E., Ortonne J.P., Ballotti R. (1998). Microphthalmia gene product as a signal transducer in cAMP-induced differentiation of melanocytes. J. Cell Biol..

[29] Buscà R., Ballotti R. (2000). Cyclic AMP a key messenger in the regulation of skin pigmentation. Pigment Cell Res..

[30] Escajadillo T., Sewer M. (2014). AKAP13 coordinates cAMP signaling and glucocorticoid production in H295R human adrenocortical cells (612.2). FASEB J..

[31] Bajova H., Nelson T.E., Gruol D.L. (2013). Chronic CXCL10 alters the level of activated ERK1/2 and transcriptional factors CREB and NF-kB in hippocampal neuronal cell culture. J. Neuroimmunol..

[32] Wagner H., Bladt S. (1996). Plant Drug Analysis.

[33] Hostettmann K., Marston A. (2005). Saponins: Chemistry and Pharmacology of Natural Products.

[34] Catalano L., Franco I., De Nobili M., Leita L. (1999). Polyphenols in olive mill wastewaters and their depuration plant effluents: A comparison of the Folin-Ciocalteau and HPLC methods. Agrochimica.

[35] Zhishen J., Mengcheng T., Jianming W. (1999). The determination of flavonoid contents in mulberry and their scavenging effects on superoxide radicals. Food Chem..

[36] Xu B.J., Chang S.K. (2007). A comparative study on phenolic profiles and antioxidant activities of legumes as affected by extraction solvents. J. Food Sci..

[37] Chen Y., Xie M.Y., Gong X.F. (2007). Microwave-assisted extraction used for the isolation of total triterpenoid saponins from Ganodermaatrum. Food Eng..

[38] Villareal M., Kume S., Neffati M., Isoda H. (2017). Upregulation of Mitf by phenolic compounds-rich *Cymbopogon schoenanthus* treatment promotes melanogenesis in B16 melanoma cells and human epidermal melanocytes. BioMed. Res. Int..

[39] Bennett D. (1989). Mechanisms of differentiation in melanoma cells and melanocytes. Environ. Health Perspect..

