# Supplementary material for: Argania Spinosa Fruit Shell Extract-Induced Melanogenesis via cAMP Signaling Pathway Activation
Source: Int J Mol Sci. 2020 Apr 6;21(7):2539. doi: 10.3390/ijms21072539 (PMC7177760; doi:10.3390/ijms21072539)
Supplement: Supplementary file 1 [file ijms-21-02539-s001.zip › Caption for Supplementary Figure S-1__IJMS-749794.docx]

**Figure S1.** Effect of argan fruit shell ethanol extract (AFSEE) on the expression level of the melanogenic enzymes, tyrosinase (TYR), tyrosinase-related protein 1 (TRP1), and dopachrome tautomerase (DCT). (A) The expression level of TYR, TRP1, and DCT was determined by western blotting. B16F10 cells were cultured in 100 mm dish at a density of 3×10^5^ cells/dish and treated without (control) or with α-MSH (200 mM) or AFSEE (6 µg/ml and 30 µg/ml) for 12 h, 24 h, 48 h and 72 h h. (B) The protein bands intensities of TYR, TRP1, DCT were obtained using Li-COR Software. Data subjected to ANOVA (n=3); different letters indicate significant difference (p ≤ 0.05).
