# Supplementary material for: Argania Spinosa Fruit Shell Extract-Induced Melanogenesis via cAMP Signaling Pathway Activation
Source: Int J Mol Sci. 2020 Apr 6;21(7):2539. doi: 10.3390/ijms21072539 (PMC7177760; doi:10.3390/ijms21072539)
Supplement: Supplementary file 1 [file ijms-21-02539-s001.zip › caption for Supplementary figure S-2_IJMS-749794.docx]

**Figure S-2.** High-performance liquid chromatography of standards used in the analysis: Chromatograms were acquired at 280 nm.
