# Supplementary figures and images for: Argania Spinosa Fruit Shell Extract-Induced Melanogenesis via cAMP Signaling Pathway Activation
Source: Int J Mol Sci. 2020 Apr 6;21(7):2539. doi: 10.3390/ijms21072539 (PMC7177760; doi:10.3390/ijms21072539)

Supplementary Figure S-2

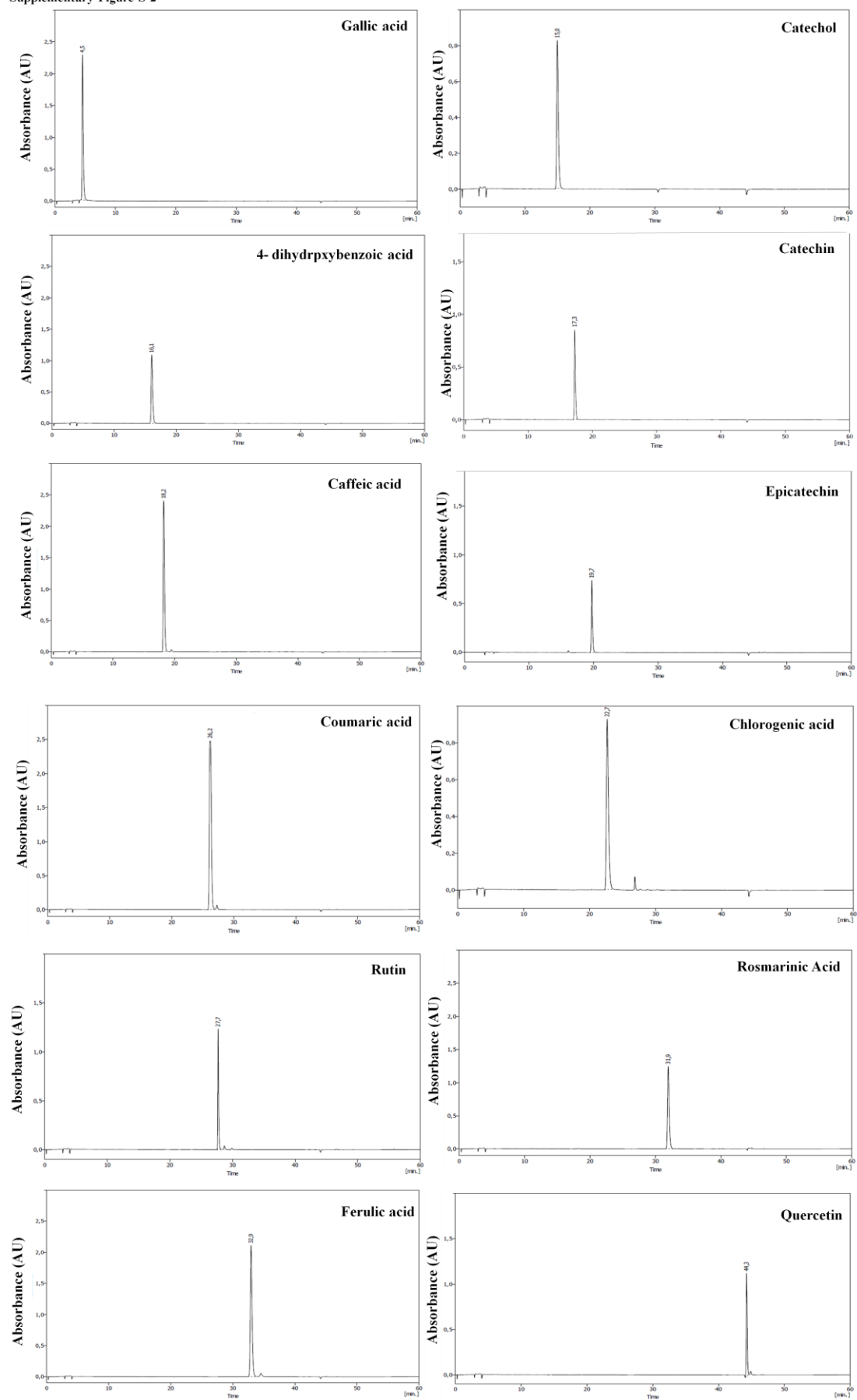

Supplement: Supplementary file 1 [file ijms-21-02539-s001.zip › Supplementary Figure S-2_Revised version_IJMS-749794.pdf]

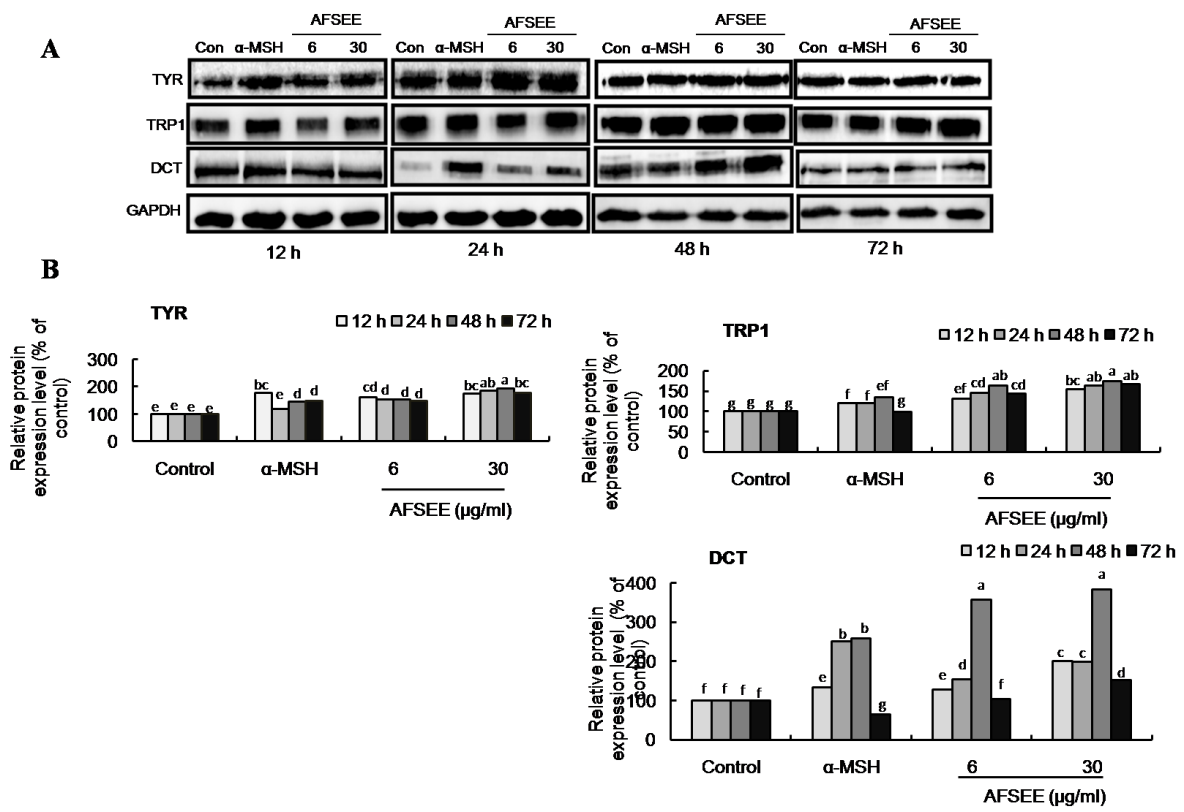

Supplement: Supplementary file 1 [file ijms-21-02539-s001.zip › Supplementary Figure S-1_Revised version_IJMS-749794.pdf]
